# Morphometric and Molecular Diversity among Seven European Isolates of *Pratylenchus penetrans*

**DOI:** 10.3390/plants10040674

**Published:** 2021-03-31

**Authors:** Mesfin Bogale, Betre Tadesse, Rasha Haj Nuaima, Bernd Honermeier, Johannes Hallmann, Peter DiGennaro

**Affiliations:** 1 Department of Entomology and Nematology, University of Florida, Gainesville, FL 32611, USA; mazene@ufl.edu; 2 Justus Liebig University, Schubertstraße 81, 35392 Gießen, Germany; betretadesse@yahoo.com (B.T.); Bernd.Honermeier@agrar.uni-giessen.de (B.H.); johannes.hallmann@julius-kuehn.de (J.H.); 3 Julius Kühn-Institut, Federal Research Centre for Cultivated Plants, Institute for Epidemiology and Pathogen Diagnostics, Toppheideweg 88, 48161 Münster, Germany; rasha.haj-nuaima@julius-kuehn.de

**Keywords:** *Pratylenchus penetrans*, *Pratylenchus fallax*, root-lesion nematode, genetic diversity, morphometrics, COXI, D2-D3 rDNA, PP5, *β*-1,4-endoglucanase

## Abstract

*Pratylenchus penetrans* is an economically important root-lesion nematode species that affects agronomic and ornamental plants. Understanding its diversity is of paramount importance to develop effective control and management strategies. This study aimed to characterize the morphological and genetic diversity among seven European isolates. An isolate from the USA was included in the molecular analyses for comparative purposes. Morphometrics of the European *P. penetrans* isolates generally were within the range of the original descriptions for this species. However, multiple morphometric characteristics, including body length, maximum body width, tail length and length of the post-vulval uterine sac showed discrepancies when compared to other populations. Nucleotide sequence-based analyses revealed a high level of intraspecific diversity among the isolates. We observed no correlation between D2-D3 rDNA- and COXI-based phylogenetic similarities and geographic origin. Our phylogenetic analyses including selected GenBank sequences also suggest that the controversy surrounding the distinction between *P. penetrans* and *P. fallax* remains.

## 1. Introduction

With a global distribution and significant economic impact [1], sometimes requiring quarantine measures [2], species within the plant parasitic nematode genus *Pratylenchus* are some of the most agriculturally important pests. Species identification within the genus is traditionally based on morphological and morphometric characterization [1,2]. The main diagnostic characteristics are presence/absence of males, body length, head shape, stylet length, and other cuticular characters including the number of lip annules, the number of lateral field lines, the presence/absence of areolated bands on the lateral fields within the vulval region, the length and structure of the post-vulval uterine sac and shape of the spermatheca, the shape of the female tail and tail tip, and de Man’s indices [3,4,5,6].

Identification and delineation of *Pratylenchus* species using these anatomical and morphometric features alone can pose many issues due to interspecific similarity and intraspecific variability of some of these characters [1,7,8]. For example, the high intraspecific morphological variations that exist within *P. penetrans* and *P. fallax* have contributed to the taxonomic confusion of these species. *P. fallax* was separated from *P. penetrans* by Seinhorst [7], only to be considered conspecific later by Tarte and Mai [8], who attributed the variations to environmental factors. The separation of the two species was confirmed using breeding experiments [9], isozyme [10] and PCR Restriction Fragment Length Polymorphism (PCR-RFLP; [11]) analyses. The presence/absence of males also does not appear to be a robust taxonomic characteristic as some asexual species such as *P. thornei, P. neglectus* and *P. hippeastri* have been reported to occasionally have males though these males may not play a role in reproduction [12]. The large number of species (110 species) described within the genus [13] is also a contributing factor owing to the limited number of distinguishing morphological features that are available. Consequently, different molecular methods have been developed for species identification and assessment of genetic variation within and between species of *Pratylenchus.* Commonly used molecular methods include quantitative PCR (qPCR; [14]), Amplified Fragment Length Polymorphism (AFLP; [15,16]), RFLP [11,17], Random Amplified Polymorphic DNA (RAPD; [18,19,20]), Sequence Characterized Amplified Region (SCAR; [16,21]), Single Nucleotide Polymorphism (SNP; [22]) and Simple Sequence Repeats or Variable Number Tandem Repeats (SSR or VNTR, respectively; [23,24]).

One of the most economically important species within this genus is *P. penetrans*, which affects a wide range of agronomic and ornamental plants, and has the potential to parasitize over 400 plant species [1,25]. *P. penetrans* is cosmopolitan though more significant in temperate regions, harbours high morphological variation, and it is considered to represent a species complex [26]. The objective of this work was to determine the diversity among seven populations of *P. penetrans* that were collected from different geographical regions in Europe based on morphometric and molecular analyses. An isolate (VA) obtained from Virginia, USA, was also included in nucleotide sequence analyses for comparative purposes.

## 2. Results

### 2.1. Morphometrical Observations

Significant similarities and differences in morphometric characters were observed amongst the seven *P. penetrans* isolates (Table 1). The ratio (b’) of body length (L) to length of pharynx (from anterior end to posterior end of pharyngeal gland) was the largest for NL, FR and UK, and the smallest for MN, WZ and BL. The ratio (c) of body length to tail length (tail) ranged from 14.10 in BN to 23.30 in FR. These isolates were significantly different from each other in terms of this ratio. The excretory pore (EP) was most anterior in MN, WZ and some UK isolates, and most posterior in BL, FR, NL and some BN isolates. Ovary length (Ovary) was significantly different between MN and BL isolates. MN and WZ isolates had shorter tails than BN, BL, FR and NL. Some morphological characters varied among the seven populations, but no distinct groupings were observed in terms of these characters. Such characters included stylet length (Stylet), pharynx length (Ph-L; anterior end to end of pharyngeal gland) and length of pharyngeal overlap (Ph-O). The distance of vulva from anterior end divided by body length (V) did not vary significantly among the seven populations.

Coefficient of variation (CV) for the various morphometric characters ranged from 2.40% to 14.92% (Table 1). CV was the lowest for Stylet length (2.40%) and a value (2.85%); and the highest for ovary length (14.92%) and length of post-vulval uterine sac (PUS; 14.59%).

### 2.2. Nucleotide Sequence Analysis

For each of the eight isolates, we sequenced the partial β-1,4-endoglucanase gene, the D2-D3 expansion of rDNA and the partial mitochondrial COXI gene region. The rDNA amplicon for each isolate was cloned (see below) and two transformed bacterial colonies were sequenced to check for the presence/absence of gene variants and/or intrapopulation variants. Both colonies that were sequenced for each isolate’s rDNA fragment had identical D2-D3 sequences. We included in our sequence alignments selected GenBank sequences spanning the D2-D3 rDNA expansion and the mitochondrial COXI sequences for which our sequences found the highest hits during nucleotide Basic Local Alignment Search Tool (BLASTn) analysis (Table 2). We also included *P. neglectus* sequences for outgroup purposes (Table 2). The aligned D2-D3 and COXI sequences (each consisting of 23 taxa, including our eight isolates; Supplementary Data S1) were analyzed as a combined dataset. The β-1,4-endoglucanase sequences were not included in the phylogenetic analyses for lack of related sequences in the public databases for use as references.

Aligned sequences were trimmed at the 5′- and 3′-ends such that nucleotide sequences including the primer sequences, or their complimentary nucleotides were excluded. This was to match the regions that we sequenced for our isolates. In the case of COXI sequences, this was also to exclude the two nucleotide differences that we observed in the middle of the JB3 binding sites (see below; indicated by boldface letters) in some GenBank sequences. In some (accession numbers MK877993–MK877996, MK877985–MK877987) the JB3 binding site had the sequence 5′-TTT TTT GG**T** CAT CC**G** GAG GTT TAT-3′, while in others (accession numbers MN453207–MN453217) this sequence was 5′-TTT TTT GG**G** CAT CC**T** GAG GTT TAT-3′. A third group of sequences (accession numbers MK877989–MK877992) had a JB3 site 5′-TTT TTT GG**T** CAT CC**A** GAG GTT TAT-3′. The D2-D3 and COXI datasets incorporated 692-and 321-characters including alignment gaps, respectively.

**Figure 1:**
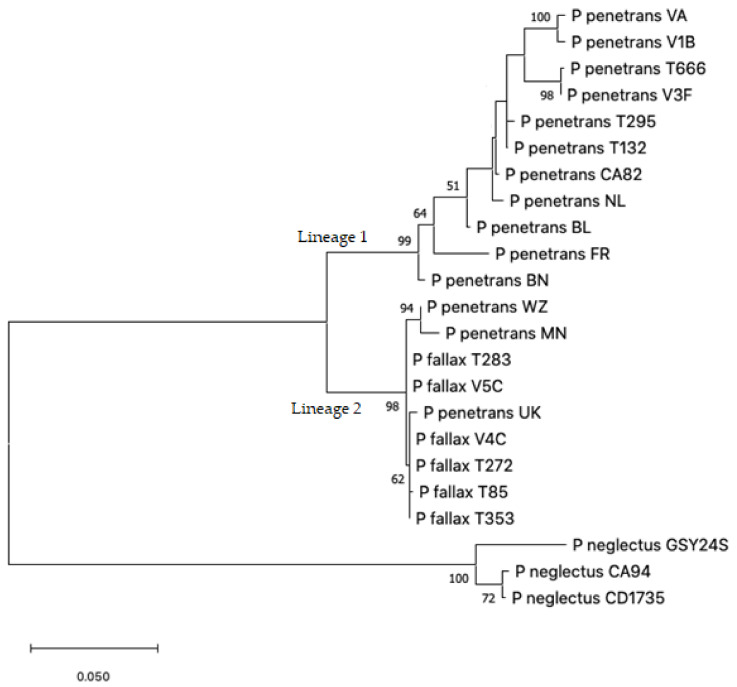
ML tree based on the combined D2-D3 rDNA and COXI dataset. Bootstrap values > 50 are shown. Scale bar indicates number of substitutions per site.

**Figure 2:**
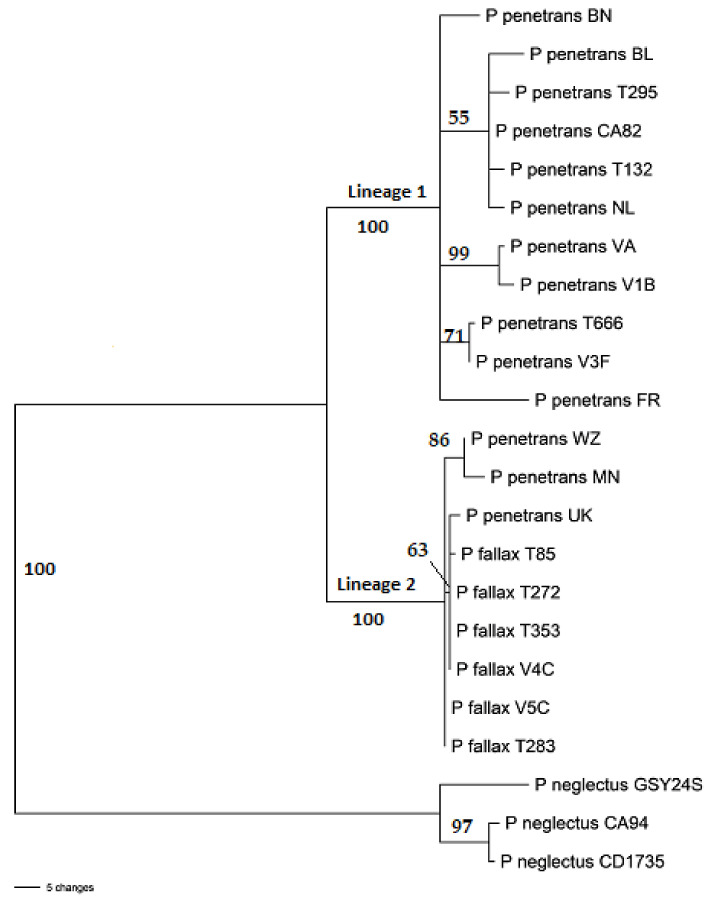
Maximum Parsimony tree generated using the combined D2-D3 and COXI dataset. Bootstrap values > 50 are indicated above nodes. Scale bar indicates number of changes.

Maximum Likelihood and Maximum Parsimony analysis of the concatenated D2-D3 rDNA and COXI dataset resulted in the trees presented in Figure 1 and Figure 2. The MP and ML trees had the same general topology though the level of bootstrap support for the two lineages and branches in these lineages differed. Both ML and MP analyses resolved the ingroup into two well-supported lineages, one of which (Lineage 2) exclusively consisted of three of our eight *P. penetrans* isolates (UK, MN and WZ) and *P. fallax* sequences from GenBank. The remaining five of our isolates fell in Lineage 1 either within well-supported groups or scattered throughout this branch. Both analyses used the General Time Reversible model [28] and all nucleotide positions were included.

## 3. Discussion

### 3.1. Morphometrical Observations

Morphometric measurements of the seven *P. penetrans* populations studied here were within the range of the original descriptions [29,30]. Most of these measurements also largely corresponded with those described for populations from China [31,32]; Colombia, Ethiopia, France, Japan, Rwanda, The Netherlands, and USA [15]; and Morocco [33]. However, remarkable differences were also observed for some characters.

Average ratios of body length to maximum body width (a) observed in the isolates examined here (25.10–27.70) were comparable to those described by Janssen et al. [15] (24.00–27.00), but lower than those reported by Chen et al. [31] (29.90–32.00) and Mokrini et al. [33] (29.20–33.00). The range of ratios of body length to pharynx length from anterior end to posterior end of pharyngeal gland (b’) in our isolates (4.33–4.98) was comparable to those described by Mokrini et al. [33] (4.40–5.00). Average body length to tail length ratios (c) ranged from 17.00 to 19.90 among our isolates. Most of these values were lower than those measured for population(s) of Wu et al. [32] (21.40), Chen et al. [31] (20.20–22.10) and Janssen et al. [13] (20.00–25.00). The *P. penetrans* isolates we studied were shorter (437–545 µm) than those described by Wu et al. [32] (666 µm), Chen et al. [31] (540–610 µm) and Janssen et al. [13] (593–684 µm). Position of the vulva relative to body length (V) in our isolates was comparable to those described by Chen et al. [31], Wu et al. [32], Mokrini et al. [33] and Janssen et al. [13]. Similarly, positions of the excretory pore (EP), maximum body width (MBW; Table 1) and tail length in the isolates we studied were comparable to those reported for other populations by Mokrini et al. [33]. Except for MBW, which was considerably higher in our isolates, EP and tail length among our isolates were also comparable to those studied by Chen et al. [31] (69.00–80.00 µm, 9.40–10.40 µm and 25.00–28.00 µm, respectively). However, measurements for these three morphometrical features were shorter in populations described by Wu et al. [32] (91.90 µm, 25.40 µm and 31.40 µm, respectively) and Janssen et al. [13] (97–120 µm, 21–28 µm and 29–32 µm, respectively). The isolates we studied had a shorter post-vulval uterine sac (PUS; 19.60–23.60 μm) than those of Mokrini et al. [33] (26.20–30.90 µm) and Wu et al. [32] (24.90 μm).

Stylet length was the least variable character among our isolates. Previous studies on *P. penetrans* [5,32] and other *Pratylenchus* species [34,35] also reported the same. This suggests that stylet length is a stable characteristic that may allow for clear demarcations among different populations of *P. penetrans* and species of *Pratylenchus*. On the contrary, ovary length and length of the post-vulval uterine sac (PUS) showed high CV among our isolates, confirming previous studies by Román and Hirschmann [5], Tarjan and Frederick [34] and Wu et al. [32]. Ph-L and Ph-O were also among the morphometric characters with high variability that we observed (Table 1). These characteristics with high CVs would be of less value in the morphological taxonomy of *P. penetrans* owing to this high variability.

### 3.2. Sequence Analysis

Mekete et al. [36] designed primer set PP5F/PP5R based on aligned *β*-1,4-endoglucanase sequences from GenBank for the purpose of identifying *P. penetrans* isolates via amplification of a species-specific 520-bp-fragment. The authors tested the primer set using isolates representing *P. penetrans, P. crenatus, P. scribneri, Helicotylenchus pseudorobustus, Hoplolaimus galeatus, Xiphinema americanum* and *X. rivesi*, where it resulted in amplification of the expected 520-bp-product only in *P. penetrans* isolates, indicating specificity of the primer set. Similarly, the authors developed a second set of primers (PSC3) that was specific to *P. scribneri* and amplified a 280-bp-fragment only in isolates of this species. In our study, PP5 amplified a PCR product in all the eight isolates. However, the size of the PP5 product among our isolates was only ~346 bp, as opposed to the expected 520 bp. BLASTn analysis of PP5-sequenes of our isolates returned *P. penetrans* β-1,4-endoglucanase as the only one or two significant match(es) from among the eight *Pratylenchus* β-1,4-endoglucanase sequences currently available in GenBank; unfortunately, Mekete et al. [36] did not sequence their PP5 PCR products. To rule out the possibility that Mekete et al. [36] confused amplicon sizes of PP5 and PSC3 in their report, we tested primer set PSC3 in our isolates. PSC3 did not produce amplification products at any of the annealing temperatures reported for this primer set [36]. While we cannot discount the usefulness of PP5 for the identification of *P. penetrans* isolates based on amplification of a PCR product, we can, however, confirm that the size of the amplicon may not always be 520 bp.

Three of our eight isolates which are grouped in Lineage 2 (UK, WZ and MN) shared several morphological characteristics apart from the remaining five isolates. The three isolates had the most anterior excretory pores, 71.60 ± 1.27 µm, 67.70 ± 1.23 µm, and 70.60 ± 1.26 µm, respectively. This was in sharp contrast to that described for *P. fallax* by Janssen et al. [13]. This measurement for *P. fallax* isolates by Janssen et al. [13] were 87 ± 8.3 µm (Ysbrechitum F2455), 91 ± 11 µm (Uddel F0689) and 108 ± 14 µm (Doornenburg–Type locality). Body and tail length in UK, WZ and MN isolates were in the short end of the spectrum for our seven isolates and matched that reported by Janssen et al. [13] for two of their *P. fallax* populations. The third *P. fallax* population (Ysbrechtum F2455), however, had much longer bodies (527 ± 32 µm). The range of pharynx length (Ph-L) reported for *P. fallax* [13] was much wider than what we found among our seven isolates. Stylet length, which showed the least variation among isolates of *P. penetrans* [this study; 6,31] and other *Pratylenchus* species [34,35], did not correlate with phylogenetic groupings. Janssen et al. [13] have attempted to resolve the controversy surrounding the separation of *P. fallax* from *P. penetrans* using morphology and sequence information. However, our findings suggest that *P. fallax* may remain to be a cryptic species along several others in the *P. penetrans* species complex [26].

Phylogenetic resolution of the seven European isolates we studied did not correspond with the geographical origins of these isolates. For example, the three German isolates that were collected not more than 40 km away from each other, grouped in two different lineages. Isolate BN grouped in Lineage 1, while isolates WZ and MN grouped in Lineage 2. On the other hand, isolates UK and WZ, which had the largest distance between their geographical origins (861 km), grouped together in Lineage 2. The isolate from the USA also grouped in Lineage 1, together with some of the European isolates, confirming that geographical origin did not correspond with phylogenetic grouping. The *P. penetrans* group [13] is known to include several more cryptic species than that represented by the two lineages here.

The separation of *P. fallax* from *P. penetrans* was based on breeding experiments that produced infertile interspecific offspring [9], and distinctive isozyme [10] and ITS-RFLP [37] patterns. We have not done any of these analyses using our isolates and cannot confirm or refute the validity of these techniques for the separation of the two species. However, the morphological variations that we observed among our Lineage 2 isolates, and the variation that Janssen et al. [13] reported among their *P. fallax* populations, taken together with the fact that MN, WZ and UK isolates grouped with *P. fallax* isolates in a strongly-supported-Lineage 2, indicates that neither morphology nor D2-D3 rDNA- and COXI-based phylogenetic analyses are sufficient to separate the two species.

## 4. Materials and Methods

### 4.1. Nematode Isolates and Microscopy

Seven of the isolates were collected from soils in different regions in Europe, multiplied from single females on carrot disc cultures for two–three generations (Table 3; [38]) and used in morphometric and molecular analyses. The eighth isolate (VA) obtained from Virginia, USA, was used in the nucleotide sequence analyses for comparative purposes.

Killing, fixing, and mounting of nematode specimens was done following Hooper et al. [39]. For each isolate, nematode suspensions were transferred into 10 mL glass vials in ~2 mL of water. A double-strength TAF fixative stock solution consisting of 10 mL formalin (35% formaldehyde), 1 mL triethanolamine and 56 mL aqua dest was prepared and heated to 70 °C in a water bath. Two mL of the hot fixative was then dispensed into each of the vials containing nematode suspensions, which were then left at room temperature for 24 h. The TAF fixative was removed from the vials leaving ~1 mL nematode suspension, which were then transferred onto 5 cm sterile plastic Petri dishes. The Petri dishes were filled with a solution consisting of 30% ethanol, 67% aqua dest and 3% glycerine, and placed in a wooden cabinet at room temperature for 5–7 weeks, covered only partially to allow evaporation. Specimens were permanently mounted in anhydrous glycerol.

The selection of morphometric characters studied was in accordance with Decraemer and Hunt [40] and Castillo and Vovlas [1]. Ten females were evaluated for each nematode sample. Measurements were performed using a Nikon ECLIPSE Ni-U microscope at 100X magnification with the aid of a Nikon DS Fi-2 camera and exclusive NIS-Elements image analysis software (Nikon, Tokyo, Japan). Morphometric data were analysed using generalized linear models using Gaussian (for homogeneous) or quasipoisson (inhomogeneous variances) families. Estimated marginal means (R version 4.0.2; [41]) were used to generate means and standard errors as well as for separation of treatments at *p* ≤ 0.05.

### 4.2. DNA Extraction

For each isolate, DNA was extracted following Holterman et al. [42] from ten nematodes (4-stage juveniles and adults). Nematodes were transferred individually into 0.2 mL PCR tubes using micropipette in a total of 25 µL. An equal volume of lysis buffer (25 µL) consisting of 0.2 M NaCl, 0.2 M Tris-HCl (pH 8.0), 1% *v/v* β-Mercaptoethanol, 0.8 mg/mL Proteinase K was then added to each sample. The tubes were briefly centrifuged at 16,000 rpm and incubated at 65 °C and 750 rpm for 2 h followed by 10 min at 100 °C in a Thermomixer (Eppendorf, Hamburg, Deutschland). Nematode lysates were used immediately or stored at −20 °C till used.

### 4.3. Nucelotide Sequence Analysis

Amplicons of ~2000 base pair (bp), ~350 bp and ~286 bp of the genes encoding for the 28S rDNA, the mitochondrial COXI gene and “PP5 region” were amplified using primer pairs 18S CL-F2 [43] and D3B [44], JB3 and JB4.5 [45], and PP5F and PP5R [36], respectively. The reaction and cycling conditions for the COXI and PP5 gene regions were as described by Bowles et al. [45] and Mekete et al. [36], respectively. These fragments were sequenced using the same primers as for the respective PCRs. The PCR cycles for the 28S rDNA consisted of an initial denaturation at 95 °C for 4 min followed by 35 cycles of denaturation at 95 °C for 45 s, annealing at 64 °C for 30 s and extension at 72 °C for 2 min; and a final extension at 72 °C for 10 min. The resulting fragments were cloned using a NEB PCR Cloning Kit (New England Biolabs Inc., Ipswich, MA, USA) following the manufacturer’s recommendations. For each isolate, two colonies were PCR-amplified using the primers supplied with the kit and sequenced using the D3B primer [44]. All amplification reactions were performed on a GeneAmp PCR System 2700 (Applied Biosystems, Thermo Fisher Scientific, Waltham, MA, USA). PCR products were purified using QIAquick PCR purification kit (QIAGEN, Germantown, MD, USA), and sequenced at Eurofins USA (https://www.eurofinsgenomics.com (accessed on 1 February 2021).

For COXI and D2-D3 rDNA gene regions sequenced in this study, selected sequences were obtained from GenBank and included here for reference and outgroup purposes (Table 2). DNA sequences generated in this study have been deposited in GenBank (Table 2). Nucleotide sequences were assembled using Geneious (Version 11.1.5, Biomatters Ltd., Auckland, New Zealand), and aligned using Clustal Omega [46], after which the alignments were manually corrected where needed using Phylogenetic Analysis Using Parsimony (PAUP, Version 4.0b 10; [47]). Maximum Parsimony (MP) and Maximum Likelihood (ML) analyses were done on the concatenated D2-D3 and COXI dataset using MEGA-X [48]. Heuristic searches based on 1000 random addition sequences and tree bisection-reconnection were used for this purpose, with the branch swapping option set on ‘best trees’ only. Bootstrap analysis [49] was based on 1000 replications.

## Figures and Tables

**Table 1:** Morphometry of the seven European *Pratylenchus penetrans* female isolates and their geographical origins.

Char.	*P. penetrans* Isolates							CV ^4^ (%)
	MN	WZ	BN	BL	UK	FR	NL	
L	449 ± 9.70 ^1^ a^2^ (431–462) ^3^	437 ± 9.60 a (381–492)	506 ± 10.30 bc (465–578)	525 ± 10.50 c (443 ± 594)	470 ± 10.00 ab (428–517)	544 ± 10.70 c (505–625)	527 ± 10.50 c (465–572)	6.23
a	26.00 ± 0.25 ab (24.80–28.60)	25.10 ± 0.25 a (22.20–28.90)	27.50 ± 0.25 d (24.40–31.20)	26.30 ± 0.25 bc (22.10–29.70)	25.10 ± 0.25 a (22.20–27.30)	27.70 ± 0.25 d (25.90–30.20)	27.10 ± 0.25 cd (24.60–30.80)	2.85
b’	4.34 ± 0.08 a (5.84–9.22)	4.38 ± 0.08 a (6.63–9.55)	4.52 ± 0.08 ab (6.32–10.30)	4.33 ± 0.08 a (5.62–8.44)	4.85 ± 0.08 bc (6.45–9.23)	4.98 ± 0.08 c (5.33 ± 9.17)	4.87 ± 0.08 c (6.92–8.50)	4.59
c	19.30 ± 0.35 bc (17.10–20.50)	19.10 ± 0.35 bc (16.40 -21.10)	17.20 ± 0.33 a (14.10–20.30)	18.20 ± 0.34 ab (14.40–20.70)	18.20 ± 0.34 ab (14.60–21.70)	20.00 ± 0.35 c (16.90–23.30)	18.40 ± 0.34 ab (16.00–21.00)	5.06
V	79.2 ± 0.81 a (77.90–80.80)	79.9 ± 0.82 a (78.60–81.90)	79.70 ± 0.81 a (73.30–82.90)	80.90 ± 0.82 a (76.80–85.90)	78.80 ± 0.81 a (76.70–81.60)	79.80 ± 0.81 a (77.30–82.70)	79.60 ± 0.81 a (76.00–82.30)	6.68
Stylet	15.10 ± 0.12 a (14.60–15.60)	15.40 ± 0.12 abc (14.50–16.00)	15.30 ± 0.12 ab (15.00–15.80)	15.80 ± 0.12 c (15.10–16.80)	15.70 ± 0.12 bc (15.20–16.40)	15.30 ± 0.12 abc (15.00–16.20)	15.20 ± 0.12 ab (14.60–15.60)	2.40
Ph-L	104.00 ± 3.50 ab (89–115)	100.00 ± 3.50 ab (90–112)	112.00 ± 3.50 bc (97–133)	121.00 ± 3.50 c (100–136)	97.00 ± 3.50 a (95–111)	111.00 ± 3.50 abc (90–161)	108.00 ± 3.50 abc (98–119)	9.20
Ph-O	37.60 ± 1.54 a (30.90–40.20)	44.10 ± 1.67 bc (37.60–50.00)	45.40 ± 1.69 bc (32.60–58.70)	46.30 ± 1.71 c (35.90–52.00)	37.30 ± 1.53 a (33.10–41.50)	40.40 ± 1.60 abc (30.50–45.80)	42.10 ± 1.63 abc (34.80–50.40)	11.33
EP	70.60 ± 1.26 a (67.10–72.40)	67.70 ± 1.23 a (58.60–72.60)	76.40 ± 1.31 bc (70.50–83.30)	81.80 ± 1.35 c (74.30–94.70)	71.60 ± 1.27 ab (69.50–74.10)	79.30 ± 1.33 c (68.20–84.30)	78.00 ± 1.32 c (73.50–82.00)	4.98
MBW	17.30 ± 0.38 a (16.00–18.00)	17.40 ± 0.38 a (16.20–19.40)	18.40 ± 0.39 ab (16.10–20.20)	19.90 ± 0.41 b (17.40–23.40)	18.70 ± 0.39 ab (17.80–20.00)	19.70 ± 0.40 b (18.00–24.00)	19.40 ± 0.40 b (16.60–21.20)	6.30
Ovary	152 ± 8.40 a (134–174)	182 ± 8.40 ab (155–218)	172 ± 8.40 ab (137–242)	191 ± 9.40 b (114–244)	163 ± 8.40 ab (131–221)	155 ± 8.80 ab (122–184)	177 ± 8.40 ab (142–220)	14.92
PUS	23.60 ± 1.04 a (18.50–29.30)	20.50 ± 1.04 a (17.40–28.50)	19.60 ± 1.04 a (15.60–26.90)	23.10 ± 1.04 a (21.30–24.40)	19.70 ± 1.04 a (17.20–23.70)	20.70 ± 1.04 a (15.70–29.30)	22.60 ± 1.04 a (18.60–26.70)	14.59
P	14.70 ± 0.40 a (1.18–2.47)	15.70 ± 0.40 a (1.06–1.72)	17.80 ± 0.40 b (0.88–1.42)	18.20 ± 0.40 b (1.14–1.55)	17.90 ± 0.40 b (0.94–1.41)	18.10 ± 0.40 b (0.83–1.45)	17.90 ± 0.40 b (1.00–1.50)	7.06
V-A	71.00 ± 2.29 ab (68.30–74.50)	66.00 ± 2.21 a (59.80–73.30)	71.30 ± 2.30 ab (64.70–80.60)	75.20 ± 2.36 abc (64.90–93.00)	77.40 ± 2.40 bc (65.50–96.5)	85.20 ± 2.51 c (70.20–107.0)	78.60 ± 2.41 bc (68.20–87.80)	9.33
Tail	23.30 ± 0.74 a (22.00–25.40)	22.80 ± 0.73 a (21.90–24.70)	29.30 ± 0.83 b (26.40–33.4)	29.00 ± 0.83 b (25.60–36.2)	26.00 ± 0.78 ab (20.30–30.40)	27.60 ± 0.81 b (23.70–31.60)	28.70 ± 0.82 b (24.70–32.10)	8.50

^1^ Average and standard error (*n* = 10), ^2^ Different letters between columns in the same row indicate significant differences according to generalized linear models and estimated marginal means with Sidak corrections for multiple comparison of means at *p* ≤ 0.05, ^3^ Range, ^4^ Coefficient of variation.

**Table 2:** Sequences used/generated in this study.

Species	Strain/Voucher	Accession Number			Reference
		D2-D3	COXI	β-1,4-endoglucanase	
*P. penetrans*	MN	MW720686	MW742327	MW737621	This study
*P. penetrans*	WZ	MW720687	MW742328	MW737622	This study
*P. penetrans*	BN	MW720688	MW742329	MW737623	This study
*P. penetrans*	BL	MW720689	MW742330	MW737624	This study
*P. penetrans*	UK	MW720690	MW742331	MW737625	This study
*P. penetrans*	FR	MW720691	MW742332	MW737626	This study
*P. penetrans*	NL	MW720692	MW742333	MW737627	This study
*P. penetrans*	VA	MW720693	MW742334	MW737628	This study
*P. penetrans*	T666	KY828351	KY816982	−	[13]
*P. penetrans*	T295	KY828352	KY816991	−	[13]
*P. penetrans*	CA82	EU130859	KY817022	−	[27]
*P. penetrans*	T132	KY828358	KY817015	−	[13]
*P. penetrans*	V3F	KY828346	KY816940	−	[13]
*P. penetrans*	V1B	KY828348	KY816942	−	[13]
*P. fallax*	V5C	KY828361	KY816937	−	[13]
*P. fallax*	T85	KY828367	KY817017	−	[13]
*P. fallax*	T283	KY828364	KY816996	−	[13]
*P. fallax*	T272	KY828365	KY816998	−	[13]
*P. fallax*	T353	KY828363	KY816988	−	[13]
*P. fallax*	V4C	KY828362	KY816938	−	[13]
*P. neglectus*	GSY24S	KY424315	KX349423	−	Unpublished
*P. neglectus*	CA94	EU130854	KU198941	−	[27]
*P. neglectus*	CD1735	KU198962	KU198940	−	[12]

**Table 3:** Isolate designation, geographical origin, and distance (km) between geographical origins of the seven European isolates.

Geographical Origin	Isolates	MN	WZ	BN	BL	UK	FR	NL
Germany (Münster)	MN	_						
Germany (Witzenhausen)	WZ	169	_					
Germany (Bonn)	BN	143	206	_				
Belgium	BL	288	428	237	_			
United Kingdom	UK	693	861	712	493	_		
France	FR	616	704	501	366	650	_	
The Netherlands	NL	129	128	127	159	594	499	_

## Data Availability

Data is contained within the article or Supplementary Materials. The data presented in this study are available in Supplementary Data S1 and sequences produced can be found in GenBank, accessions listed in Table 2.

## Supplementary Materials

The following are available online at https://www.mdpi.com/article/10.3390/plants10040674/s1, Data S1: D3 rDNA and COXI sequences of 37 taxa (including our eight isolates) still groups MN, WZ and UK isolates in a strongly supported branch together with *P. fallax* isolates.
